# The role of erythropoietin-loaded hydrogel versus adipose derived stem cell secretome in the regeneration of tongue defects

**DOI:** 10.1186/s12903-024-04835-0

**Published:** 2024-09-18

**Authors:** Rana El-Qashty, Jilan Youssef, Eman Hany

**Affiliations:** 1 Oral Biology department, Faculty of Dentistry, Mansoura University, Mansoura, Egypt; 2 Periodontology, Diagnosis and Oral Radiology Department, Faculty of Dentistry, Oral Medicine, Mansoura University, Mansoura, Egypt

**Keywords:** Tongue diseases, Inflammation, Adipose tissue, Mesenchymal stem cells, Conditioned media, Anti-inflammatory M2 macrophage, CD163

## Abstract

**Background:**

Tongue defects have several etiologies and significantly affect the quality of life. This study was conducted to compare the regenerative potential of erythropoietin (EPO)-loaded hydrogel and adipose derived stem cell (ADSC) secretome on tongue dorsum defects focusing on the role of anti-inflammatory M2 macrophage phenotype.

**Methods:**

Rats were subjected to induction of mechanical circular defects on the dorsal surface of the tongue, then divided into three groups; Group I (control): received 0.1 ml phosphate buffered saline, Group II (EPO): received 5000 U/kg EPO-hydrogel, and Group III (ADSC-Secretome): received 0.1 ml ADSC-Secretome. Treatments were injected circumferentially around wound margins after induction. Seven and fourteen days after treatment, specimens were obtained and processed for histological and immunohistochemical staining followed by the relevant histomorphometric and statistical analyses.

**Results:**

Seven days after treatment, groups II and III presented defects with some epithelial regeneration at the lateral margins, while the center of the defect showed granulation tissue with much inflammatory cells. The base of the defects showed some muscle fibers and new blood vessels, however group III showed more enhanced neovascularization. Fourteen days after therapeutic intervention, group II defects were completely covered with epithelium showing a thin keratin layer with regular rete pegs interdigitating with the underlying connective tissue papillae, but tongue papillae were not restored. Group III expressed much better healing with developing filiform papillae. The connective tissue showed more vascularity and well-arranged muscle bundles. Both treated groups showed a significant decrease in defect depth and significant increase in anti-inflammatory macrophages compared to the control group at both time intervals, however there was no significant difference between the two treated groups.

**Conclusion:**

Both treatments showed promising and comparable results in the treatment of tongue defects reducing inflammation and restoring tongue histological architecture with significant upregulation of M2 macrophage.

## Background

The tongue is a strong muscular organ comprised of an external mucosal membrane formed of stratified squamous epithelium with numerous lingual papillae carrying taste buds, and overlying an ecto-mesenchymal connective tissue, surrounding a combination of intrinsic and extrinsic striated muscles [1]. Tongue defects can result from several etiological factors including trauma, recurrent ulcers, and surgical excision of the diseased tissue [2] such as erythroplakia, squamous cell carcinoma, granular cell tumor, and kaposi sarcoma that significantly complicate speech, swallowing, and respiration [3, 4]. Up till now, there has been no specific treatment that thoroughly restores the anatomy and function of the tongue in patients suffering from tongue defects.

Hydrogels represent a widely used drug-delivery system. They are biological materials manufactured through a physico-chemical monomer crosslinking reaction, forming a polymer network structure [5]. They have numerous advantages including ease of preparation and use, minimal toxicity, and low cost [6]. More specifically, thermosensitive and injectable hydrogels have the capability to fill up the minute tissue spaces simply by injecting the pre-gel solution. They can encapsulate medications, cells, inorganic minerals and other therapies transmitting them to the targeted site, providing a long-lasting release and effective drug delivery system, but they do not share in healing tissues or repairing defects by themselves [7].

Chitosan (CS) and beta-sodium glycerophosphate (β-GP) based hydrogels are one of the most widely used hydrogels in the biomedical field. These two components have excellent biocompatibility and successfully form a hydrogel at body temperature [8]. Gelatin (a kind of ampholyte) incorporation is also recommended to crosslink CS and β-GP via an electrostatic interaction between anions and cations to reduce the gelation time [9]. All these advantages allow the quick formation of the hydrogel in the irregular tissue defects in-vivo for sustained drug release [10].

Erythropoietin (EPO), a member of the hematopoietic class I cytokine superfamily, is a 34 kDa glycoprotein hormone that maintains normal red blood cells’ count through the regulation of the precursor cells’ proliferation and differentiation and prevention of bone marrow erythroid cells apoptosis [11]. However, EPO receptors have also been detected in cells other than hematopoietic ones such as cells of the nervous system [12], cardiovascular system [13], as well as the basal cells of oral mucosa [14]. EPO showed high potentiality for regeneration of different oral tissues as tongue ulcers [10], and treatment of gingival and periodontal diseases [15–18].

Erythropoietin enhances tissue regeneration following damage via several principal mechanisms. It exerts an anti-inflammatory effect by inhibiting the activity of inflammatory cells and suppressing the release of proinflammatory cytokines such as interleukin-1β (IL-1β), interleukin-6 (IL-6), tumor necrosis factor-alpha (TNF-α), reactive oxygen species (ROS), and membrane lipid peroxidase [19]. Recently, it has been found that EPO can modulate the immune system bioactivities [20]. Specifically, EPO can enhance the phagocytic capacity of macrophages and facilitate macrophage polarization [21]. EPO has the ability to suppress the proinflammatory M1 macrophage and amplify the anti-inflammatory M2 phenotypes thereby favoring the macrophages immunoregulatory switching by activating a sequence of signaling pathways including mitogen-activated protein kinase (MAPK), phosphoinositide 3 kinase (PI3K), and Janus-tyrosine kinase 2 (Jak2)/signal transducer and activator of transcription (STAT) pathways [22].

EPO also has the capacity to induce more effective tissue restoration by accelerating the formation of granulation tissue during wound healing and its subsequent remodeling [23]. Moreover, it enhances angiogenesis by activating vascular endothelial growth factor (VEGF) which interacts with EPO inducing the formation of new functioning capillaries [24, 25]. In addition, it induces erythrocytes and leukocytes proliferation and differentiation within the defect through acceleration of cellular growth and enhancement of tissue oxygenation [26].

Stem cells are currently gaining a lot of attention as an innovative therapy to enhance the healing of tongue defects. Mesenchymal stem cells (MSCs) are multipotent stromal cells existing in most tissues. They have the capacity to differentiate into multiple cell lineages; adipogenic, chondrogenic and osteogenic. They can escape allogeneic immunogenic reactions and have high potentiality for immunomodulation and for suppressing inflammation [27].

Adipose derived stem cells (ADSCs) are isolated from adipose tissue and can enhance angiogenesis and wound regeneration in soft tissue defects. They have the advantages of being widely available, easily harvested in large number, and relatively easily expanded in culture for clinical use [28]. Even though tremendous advancements have been achieved in the field of stem cell therapy, numerous limitations still need to be addressed. These limitations include poor cell survival, limited cell availability, and even the possibility of tumorigenic transformation [29]. In addition, cells have large molecular size that may result in their entrapment in the pulmonary blood vessels [30].

Stem cell-derived secretome is the reservoir of all the biological factors and molecules that are secreted by the cells and play fundamental roles in many cellular functions such as angiogenesis and immunomodulation in addition to their anti-inflammatory and anti-apoptotic effect [31]. It principally comprises two main constituents, which are soluble factors such as cytokines, growth factors, and enzymes, besides the extracellular vesicle compartment containing proteins, lipids, and nucleic acids [32], and ranging in size from as small as 30 nm to as large as 10 μm [33]. It represents a potential novel cell free therapeutic strategy to manage orofacial soft tissues defects based on the fact that stem cells mediate wound healing mainly via their paracrine mechanisms [34]. Stem cell secretome therapy has revealed promising results in regenerative medicine including hepatic, respiratory and neurological tissue regeneration [35], as well as in wound healing [36, 37], arthritic diseases [38, 39], and immune-mediated diseases [32].

ADSCs release a wide array of growth factors for instance; basic fibroblast growth factor (bFGF), transforming growth factor- beta (TGF-β), keratinocyte growth factor (KGF), hepatocyte growth factor (HGF), and VEGF into their culture media, which mediate the wound-healing effect of ADSCs [40]. Therefore, ADSC-Secretome have comparable capacity to their parent cells to regenerate soft tissue defects with the added advantages of being relatively easy to separate, store and deliver, overcoming the problems of immunogenic rejection and the possibility of tumorigenesis representing an ideal alternate for stem cell based therapy holding a better future for soft tissue therapy [41].

Macrophages play a major role in host immunologic reaction and tissue homeostasis, through identification of various pathogens, their phagocytosis, induction and relief of inflammation, immune regulation, and initiation of tissue-restoration [42]. Their phenotype switches between the pro-inflammatory M1 and the anti-inflammatory M2 according to the signals they receive from their environment [43]. A variety of macrophage-related surface markers can be used to identify their phenotypes which also function in initiating, progressing, and relieving inflammation [44]. M1 subtype expresses CD38, CD86, CD80, formyl peptide receptors (Fpr2), G protein (Gpr18), toll-like receptors (TLR-2, TLR-4) and TNF-α, while M2 subtype expresses CD163, CD206, c-Myc and early growth response gene-2 (Egr2) [45].

To our knowledge no previous studies have compared the regenerative effect of EPO-hydrogel versus that of ADSC-Secretome on tongue defects. So, this study aimed to compare the potentiality of EPO-hydrogel to that of ADSC-Secretome in healing of induced tongue dorsum defects in rat models and explore their effect on anti-inflammatory M2 macrophage.

## Materials and methods

### Animals

Animal handling was performed in accordance with the guidelines of Mansoura University animal care and use committee with code number: MU-ACUC (DENT.R.24.05.9) and following the Animal Research: Reporting of In Vivo Experiments (ARRIVE) guidelines.

Forty adult, male, healthy, Sprague Dawley rats, aging 10–11 weeks old, and ranging in weight between 250 and 300 g were purchased from the medical experimental research center (MERC) (Mansoura University, Mansoura, Egypt) where all the experimental work was undertaken. Rats were kept in cages measuring 20 cm height and 40 cm length, 3 rats in each cage. The cages were kept in a 26 °C controlled temperature room, with approximately 65–70% humidity, and a light/dark cycle of 12 h.

Rats had free access to commercial diets (fodder containing 23% protein) and water *ad libitum*. Total and routine medical care and check-ups were applied during the study. All the biosecurity and biosafety procedures were performed following the recommendations of McCormick-Ell J and Connell N [46] where cages were kept in specified rooms, with self-closing doors, impervious and sanitizable surfaces, and handwashing facilities, separated from other animal strains, with only well-trained personnels allowed to access the rooms. Protective laboratory coats, gowns, and protective eyewear were used during surgical procedures to avoid any possible splashes. Double gloves were worn to protect hands during all steps of animal handling.

Animals were anesthetized before defect preparation and administered an analgesic and antibiotic for three days thereafter. Anesthetic overdose was used for animal euthanization, then plastic bags were utilized for wrapping the carcasses before careful transportation to an incinerator for burning.

### Erythropoietin loaded CS/β-GP/gelatin hydrogel preparation and characterization

Erythropoietin-loaded hydrogel preparation was performed following a previous protocol by Xu et al. [47]. In brief, chitosan nanoparticles were prepared by dissolving 40 mg of chitosan in 20 mL of acetic acid (0.1%) under agitation. For gelatin crosslinking, 1 mL deionized water was used for liquefaction of 5 mg gelatin at room temperature. 0.22 μm syringe filter was then used for filtering the resulting solutions. After that, 2.8 mL tripolyphosphate (TPP) solution (0.1% W/V) was used for dissolving 1.02 g of β-GP followed by filtering using a 0.22 μm syringe filter.

For EPO loading, 2.21 mL of EPO (ATC code: B03XA01, Janssen-Cilag Ltd, High Wycombe, Bucks, UK, 10000 U/mL) was added to the prepared solutions and when a uniform mixture was obtained, a solution of sodium hydroxide (NaOH) (0.1 mol/L) was added dropwise until a 7.0 pH was reached. Finally, the assembled EPO loaded CS/β-GP/Gelatin hydrogel was incubated at 37 °C for 5 min.

For characterization of the prepared EPO-hydrogel, particle size and distribution, as well as Zeta potential were analyzed using dynamic light scattering (DLS) (Zetasizer Nano ZN, Malvern Panalytical Ltd, United Kingdom) at fixed angle of 173° at 25 °C, where analysis was performed in triplicates. The average measurement of the particle size was 349.5 ± 12.45 nm, whereas Zeta potential recorded an average of 11.2 ± 0.90 mV.

### ADSCs isolation

ADSCs were isolated from six, healthy, Sprague Dawley rats weighing 200–250 g (8–9 weeks of age) per our previous protocol [48]. Briefly, the adipose tissue of rats’ supra-renal fats was collected and washed thoroughly. The collected tissues were minced into small pieces, followed by digestion in 0.1% type I collagenase (cat. #SCR103, EMD Millipore Corp, Billerica, USA), at 37 ⁰C for one hour. To stop the digestive action of the added enzyme, Dulbecco’s modified eagle medium (DMEM) culture media (cat. #L0066-500, BioWest, Nuaillé, France) complemented with 10% fetal bovine serum (FBS) (cat. #S1810-500, BioWest, Nuaillé, France) was added. Cells were then incubated at 5% CO_2_ and 37 ⁰C and the media was changed every 2–3 days thereafter. Cells were examined on daily basis using an inverted microscope (Olympus, CKX41SF, Tokyo, Japan) until 80% confluence was confirmed.

### ADSCs characterization

The flowcytometric immunophenotype characterization of ADSCs was carried out using direct staining technique in a BD Accuri C6 flow cytometer (BD Biosciences, California, USA) and BD Cell Quest ^TM^ Pro version 6.0 program software. Third passage cells were trypsinized, washed with phosphate buffered saline (PBS) and incubated with the primary antibodies; anti-CD90 PE (BD Biosciences, cat. #551401, California, USA), anti-CD73 purified (BD Biosciences, cat. #551123, California, USA), anti-CD34 purified (R&D systems, cat. #AF6518-SP, Minneapolis, USA), and anti-CD45 FITC MAB (BD Biosciences, cat. #561867, California, USA). Fluorescein isothiocyanate (FITC, cat. #F143, Thermo Fisher Scientific, Massachusetts, USA) was added to the purified antibodies as a fluorophore, then incubated in the dark at 4 °C for 30 min. Labeled ADSCs were rinsed in PBS, then centrifuged for 5 min at 200×g, followed by their resuspension in PBS.

### ADSC-Secretome preparation

ADSC-Secretome was prepared following our previous protocol [38] where third passage ADSCs (4 × 10^6^) were collected after reaching 80–90% confluence, washed three times with PBS and then cultured in FBS-free media for 48 h. After that, the media was collected, centrifuged, and then the supernatant was filtered through a 0.2 μm filter and stored at -80 °C.

### Surgical preparation of mechanical tongue defects

**Fig. 1:**
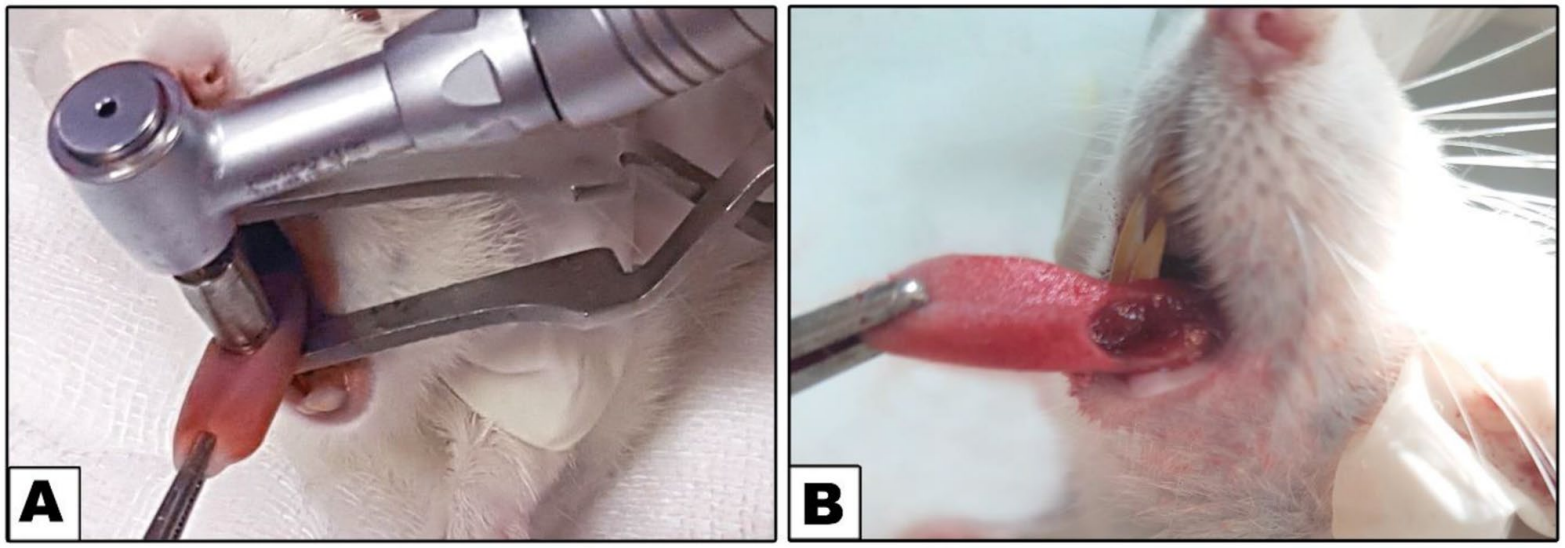
**A.** Tongue dorsum surgical defect preparation, **B.** Circular tongue defect

For the adjustment of different drugs’ administration doses, each rat (10–11 weeks old) was weighed before any surgical intervention. Animals were anesthetized using 5–7 mg/kg xylazine hydrochloride (ADWIA, 5th Settlement New Cairo, Egypt) and 35–45 mg/kg ketamine hydrochloride (Pfizer, NY, USA) intramuscular injection. Betadine was used for wiping rats’ tongues before defect preparation. Following our previous protocol [41], the defects were prepared in the median line of the dorsal surfaces of the tongues, in the middle thirds. A tissue punch centered 5.0 mm rotary (cat. #N-1042B, OrthoSourceglobal, North Hollywood, CA 91605, USA) was used for preparation of defects measuring 5 mm in diameter and 2 mm in depth **(**Fig. 1). Surgical scissors were then used to incise the tissues. Instantly following defect preparation, the tested therapies were administered through injection at the peripheries of the wound. As a postoperative antibiotic and analgesic treatment, rats received a daily dose of oxytetracycline hydrochloride 20% and analgin 0.5 mg via intraperitoneal injection for three days thereafter.

### Study design

This was an experimental, randomized controlled study. Calculation of the sample size was performed based on a previous study depending on the average ulcer depth in the control and EPO groups after 14 days [10]. Calculations were performed through G*power version 3.1.9.4 with effect size of 2.5458, 2-tailed test, α error 0.05, and power 95.0%. The size was found to be at least 6 in each group.

Four rats were euthanized at the start of the experiment to evaluate the normal histology of rat’s tongue. The remaining rats were subjected to induction of mechanical tongue defect and then allocated to three different groups (*n* = 12) following a simple random sampling method according to the type of treatment that was injected circumferentially around the wound margins immediately after defect preparation as follows:

#### Group I (control)

Tongue defects received 0.1 ml PBS.

#### Group II (EPO hydrogel)

Tongue defects received a single dose of EPO hydrogel (5000 U/kg) [10, 49].

#### Group III (ADSC-Secretome)

Tongue defects received 0.1 ml ADSC-Secretome [41].

Intraperitoneal injection of anesthetic overdose (120 mg/kg thiopental sodium, alfa chemical group, Cairo, Egypt) was used for euthanasia [50]. Out of each group, six rats were euthanized at 7 and 14 days after defect induction. Tongue samples were obtained and processed for histological and immunohistochemical investigations.

### Histological and immunohistochemical staining

After collection and fixation of tongue specimens in neutral-buffered formalin, they were processed to prepare paraffin blocks. Using a microtome, 4 μm tissue sections were serially cut, followed by their deparaffinization, rehydration, and staining. Hematoxylin and eosin staining (H&E) was performed for monitoring the histological changes and evaluating the regenerative process through the healing period.

Immunohistochemical staining was performed for detection of anti-inflammatory M2 macrophage phenotype through CD163 marker (Recombinant Anti-CD163 antibody, cat. # ab182422, Abcam, Cambridge, UK, dilution 1:500). Briefly, blocking of endogenous peroxidase was carried out using H_2_O_2_, followed by antigen retrieval through boiling in citrate buffer, then incubation with CD163 primary antibody. After that, slides were incubated with the secondary biotinylated antibody, followed by streptavidin biotin complex. Diaminobenzidine chromogen (DAB substrate kit, cat. # ab64238, Abcam, Cambridge, UK) was then added followed by counterstaining with Harris hematoxylin.

### Digital histomorphometrical image analysis

H&E-stained tissue sections were examined and photographed using a digital camera (ToupCam^®^, model no. XCAM1080PHA) mounted on light microscope (Olympus^®^, CX22, Japan), with 0.5 photo adaptor, using 10x objective lens. For immunohistochemically stained sections, five different sites (1 × 1 mm^2^) in each slide were assessed through a 20x objective lens by two blinded examiners.

For digital image analysis, image processing software Fiji ImageJ (version 2; NIH, Maryland, USA) was utilized. For vertical defect depth measurements, the ulcer depth was calculated for each slide using the measuring function after image calibration where three readings were recorded from each slide from the deepest points in the defects to the top of surface epithelium at wound margins. For immunohistochemical stained sections, the percentage of positive brown staining area to the total area was calculated regardless of stain intensity where the function color deconvolution 2 tool was used to digitally separate the histological dyes in the microphotographs resulting in three digital images (a complementary image, H&E, and DAB), after which the stain-specific area percentage in DAB and H&E images was calculated. Data was presented as the mean of the vertical depth or positive brown staining percentage ± standard deviation.

### Statistical analysis

Data was analyzed using GraphPad Prism 9 (GraphPad Software). The normality of data was tested using the Shapiro-Wilk test. Normally distributed quantitative data was presented as mean ± standard deviation. Significance of the acquired results was judged at the 0.05 level. The two-way ANOVA test was used to assess the combined effect of time and intervention independent factors on the dependent continuous outcomes which were defect depth, and CD163 antibody immunostaining using Post Hoc Tukey test for pairwise comparison.

## Results

### ADSCs characterization results

**Fig. 2:**
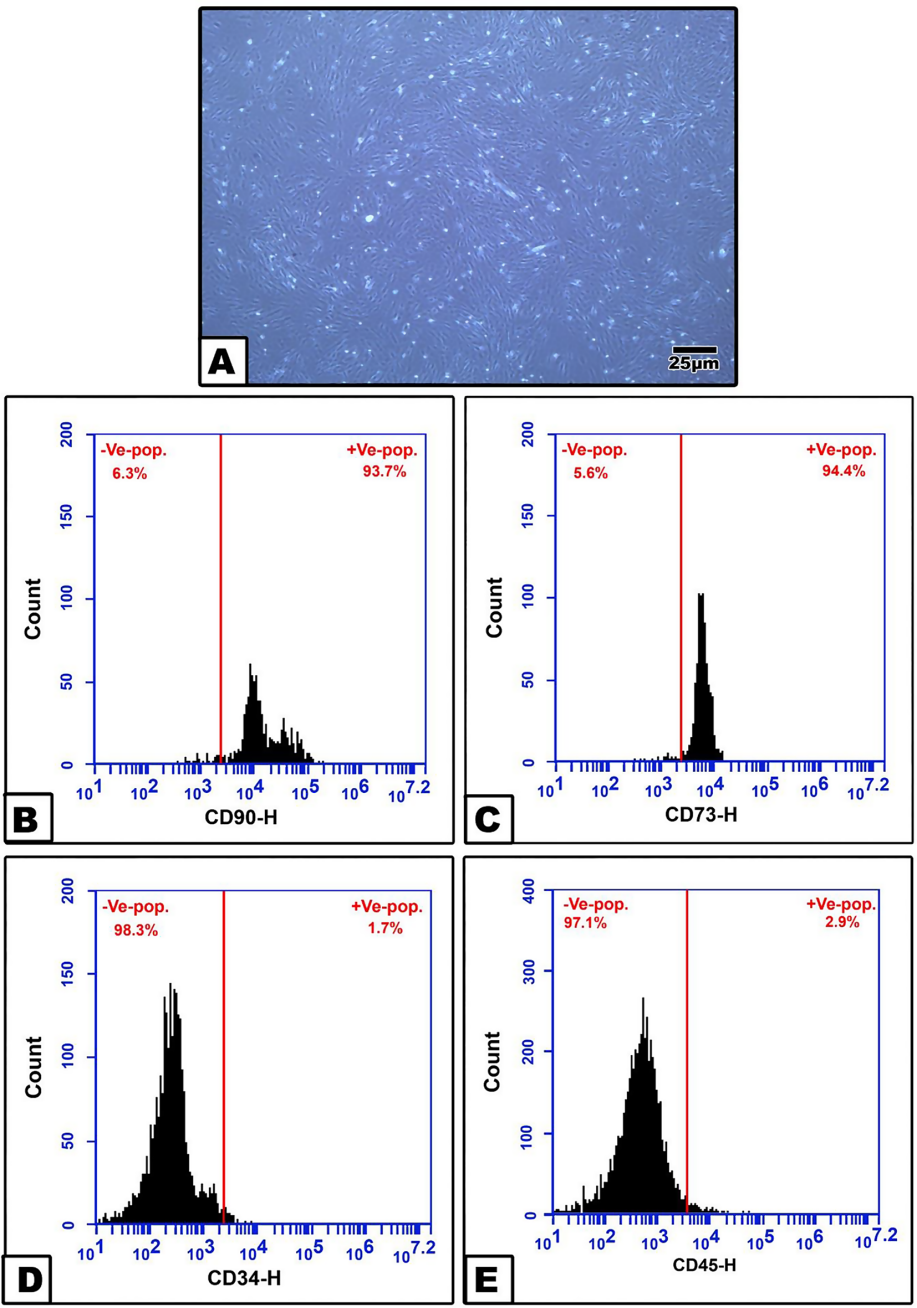
**A.** ADSC confluence. Flowcytometry histograms for ADSC phenotypic characterization. ** B.** CD90, ** C.** CD73, ** D.** CD34, ** E.** CD45

ADSCs adhered to plastic walls with typical fibroblast-like and spindle shaped morphology (Fig. 2A). The third passage ADSCs revealed highly positive results for the phenotypic cell surface mesenchymal markers CD90 (93.7%) and CD73 (94.4%) and negative results for the hematopoietic markers CD34 (1.7%) and CD45 (2.9%). (Fig. 2B-E)

### H&E results

**Fig. 3:**
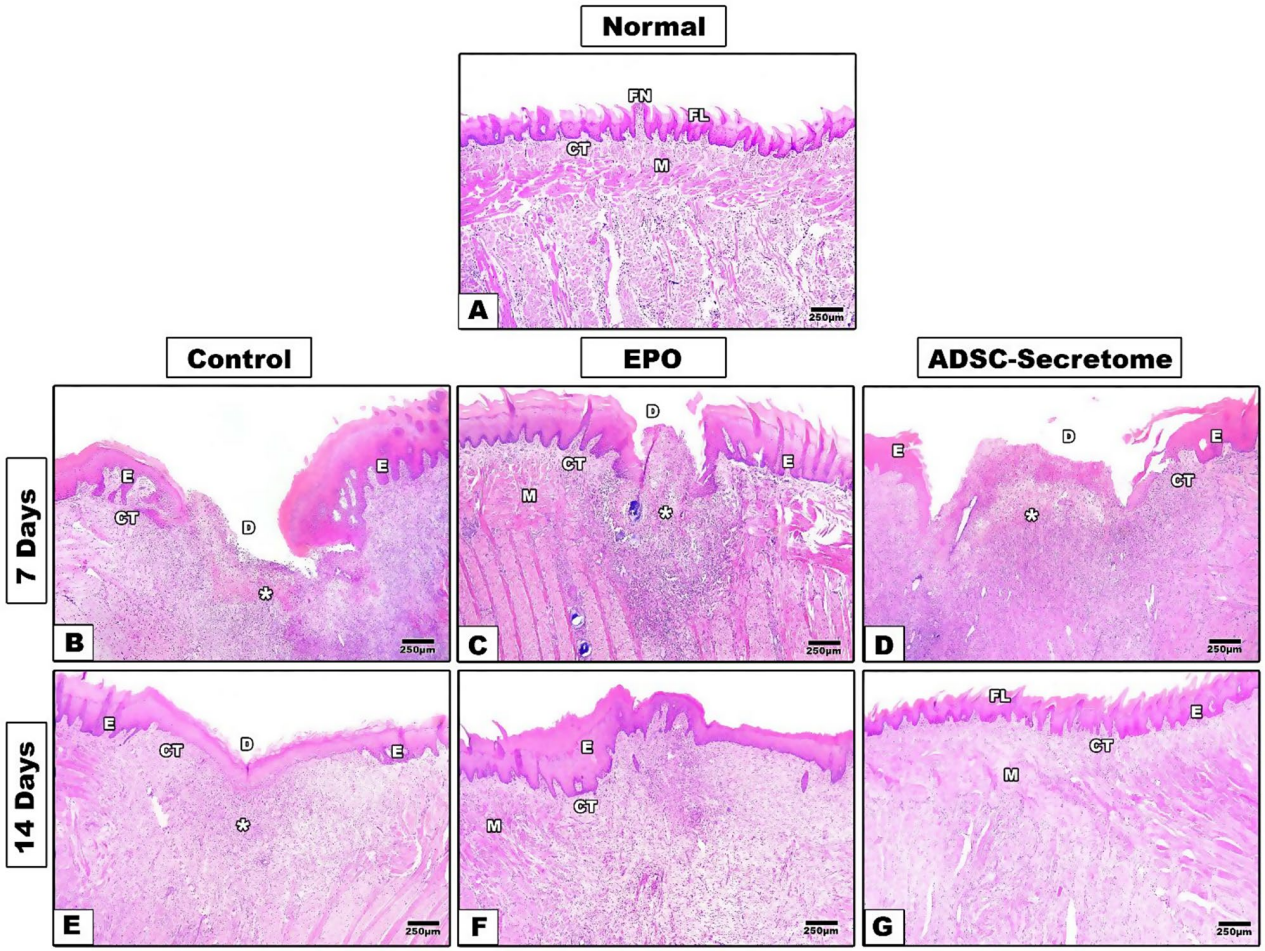
Hematoxylin and Eosin staining results of tongue specimens of different groups (4x) **A.** normal tongue, **B**,** E.** control group, **C**,** F**. EPO-hydrogel group, and **D**,** G**. ADSC-Secretome group. **E**: epithelium, **CT**: connective tissue, **M**: muscles, **D**: defect, **FL**: filiform papilla, **FN**: fungiform papilla, **Asterisk**: inflammatory infiltrate

**Fig. 4:**
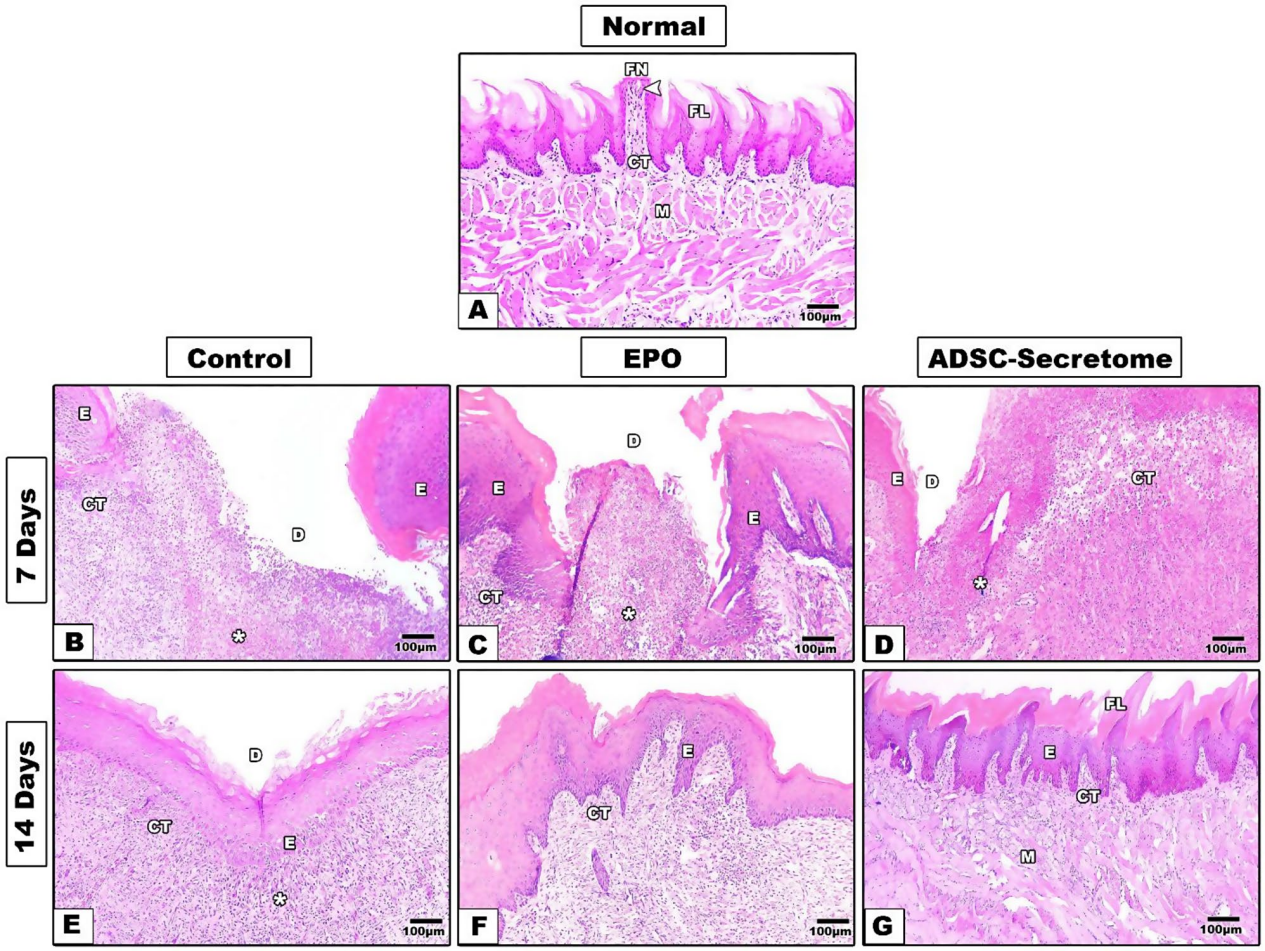
Hematoxylin and Eosin staining results of tongue specimens of different groups (10x) **A.** normal tongue, **B**,** E.** control group, **C**,** F**. EPO-hydrogel group, and **D**,** G**. ADSC-Secretome group. **E**: epithelium, **CT**: connective tissue, **M**: muscles, **D**: defect, **FL**: filiform papilla, **FN**: fungiform papilla, **Asterisk**: inflammatory infiltrate, **Arrowhead**: taste bud

The normal tongue specimens showed regular tongue histology with full thickness epithelium covered by a uniform even keratin layer. Filiform papillae covered the dorsum of the tongue with some intervening fungiform papillae carrying taste buds. The lamina propria showed regular collagen bundles with proper interdigitation with the epithelial rete pegs, and a highly arranged muscular system was observed with different muscle bundles’ orientation. (Figures 3A and 4A)

**Fig. 5:**
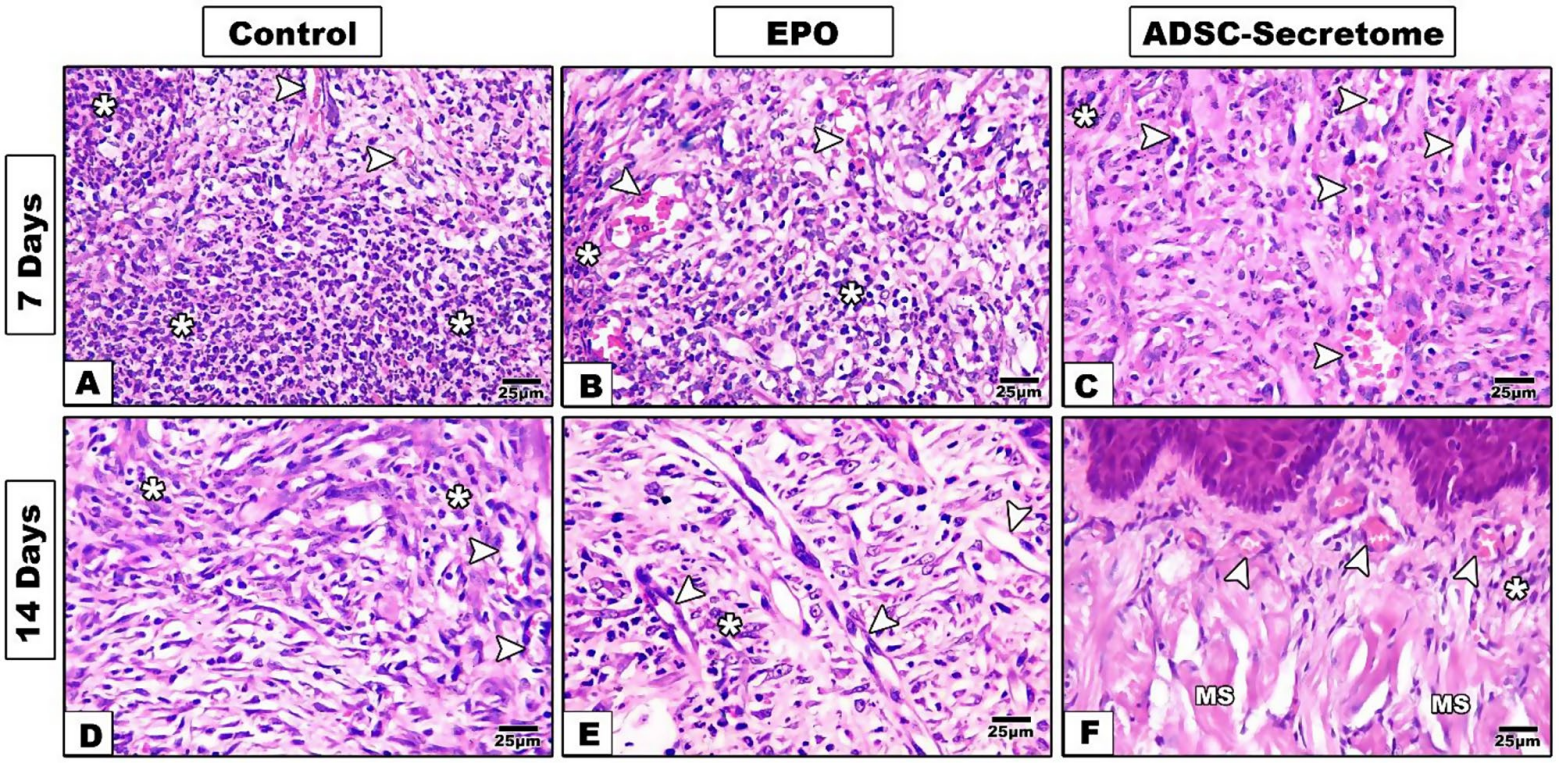
Hematoxylin and Eosin staining results of tongue specimens of different groups (40x) **A**,** D.** control group, **B**,** E**. EPO-hydrogel group, and **C**,** F**. ADSC-Secretome group. **Asterisk**: inflammatory infiltrate, **Arrowhead**: blood vessel, **MS**: muscles

Seven days after mechanical defect induction and different interventions, the control group specimens showed wide and deep defects with complete loss of epithelium and connective tissue at the defect site. Granulation tissue with much inflammatory cell infiltrate was observed. The lateral wound margins presented some irregular re-epithelization and no signs of muscular regeneration in the deep underlying tissues was detected. (Figures 3B, 4B and 5A)

In the EPO treated group, the wounds seemed smaller in size, and the lateral margins showed some epithelial regeneration with some keratin covering. Moreover, the base of the defects started to show some epithelial re-organization, and some muscle fibers. However, much granulation tissue was still found with many inflammatory cells, and little neovascularization was detected. (Figures 3C, 4C and 5B)

In the ADSC-Secretome treated group, some regeneration was also detected. Granulation tissue with inflammatory cells were observed in the wound site. More new blood vessels were formed, and the lateral margins also presented developing epithelial tongues with keratin coverage. Some muscle fibers were detected in the deep connective tissue. (Figures 3D, 4D and 5C)

Fourteen days after induction, defects of the control group still showed some deepening from the surface level. However, complete epithelial coverage was observed, but with thin, irregular keratin layer and no tongue papillae. The connective tissue expressed good healing with some blood vessels and some muscle regeneration at the depth of the defect. (Figures 3E, 4E and 5D)

The EPO-treated group showed much better regeneration as compared to the control group and to the same group at seven days. Mucosal ulceration disappeared in most tongue specimens and the surface was completely covered with epithelium showing a thin keratin layer. Regular rete pegs were observed interdigitating with the underlying connective tissue papillae. The lamina propria showed collagen bundles with new blood vessel formation. More muscle bundle re-organization was detected at the depth of the defect. However, tongue papillae were not restored through the whole defect area. (Figures 3F, 4F and 5E)

Specimens of the ADSC-Secretome treated group expressed much better healing with more regular epithelial and keratin layers with uniform thickness. Developing filiform papillae were observed. The connective tissue showed more arranged collagen bundles and more vascularity. Well-arranged muscle bundles were detected as well. (Figures 3G, 4G and 5F)

### Histomorphometric results

**Table 1:** Post Hoc Tukey test for pairwise comparison of factors affecting defect depth (µm)

Intervention Time	Control	EPO	ADSC-Secretome
**7 days**	482.42 ± 81.03	206.82 ± 43.21 ^**a**^	139.36 ± 21.01 ^**a**^
**14 days**	321.19 ± 22.64 *	53.39 ± 10.10 ^a^*	41.15 ± 4.67 ^a^*

Data presented as Mean ± SD, Significance at *p* < 0.05

^**a**^:Significance Vs control group within the same time point

*: Significance Vs 7 days within the same group

**Fig. 6:**
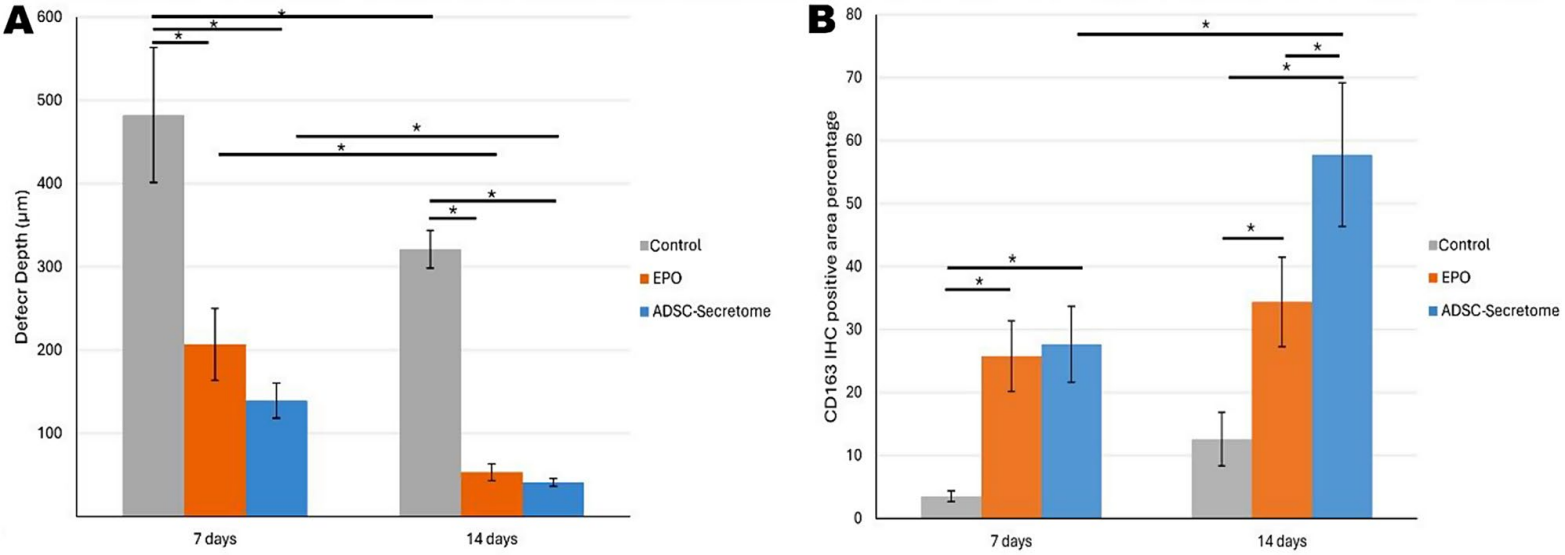
Bar graphs showing the two-way ANOVA statistical analysis for: **A.** defect depth, **B.** anti-CD163 immunohistochemical staining results. **Asterisk**: denotes statistical significance

Defect depth analysis confirmed the therapeutic influence of both treatments on tongue ulcers, where the defect measurements were very much reduced (Table 1) (Fig. 6A). The two-way ANOVA analysis showed insignificant interaction between the intervention and the time factors on the tongue defect depth [F [2, 24] = 1.862, *p* = 0.1771], therefore main effect analysis of each factor was reported.

As regards the intervention factor, a significant main effect was detected [F [2, 24] = 181.4, *p* < 0.0001], where the EPO and ADSC-Secretome groups showed significantly reduced defect depth as compared to the control group at both time intervals (*p* < 0.0001). However, both treatment groups didn’t differ significantly at 7 days (*p* = 0.1168), or at 14 days (*p* = 0.9962).

As for time factor, a significant main effect was also expressed [F [1, 24] = 89.62, *p* < 0.0001]. The pairwise comparison showed significantly lower values for all groups at 14 days’ time point versus 7 days with *p* values of < 0.0001, < 0.0001, and 0.0079 for the control, EPO, and ADSC-Secretome groups, respectively.

### Immunohistochemical results

**Fig. 7:**
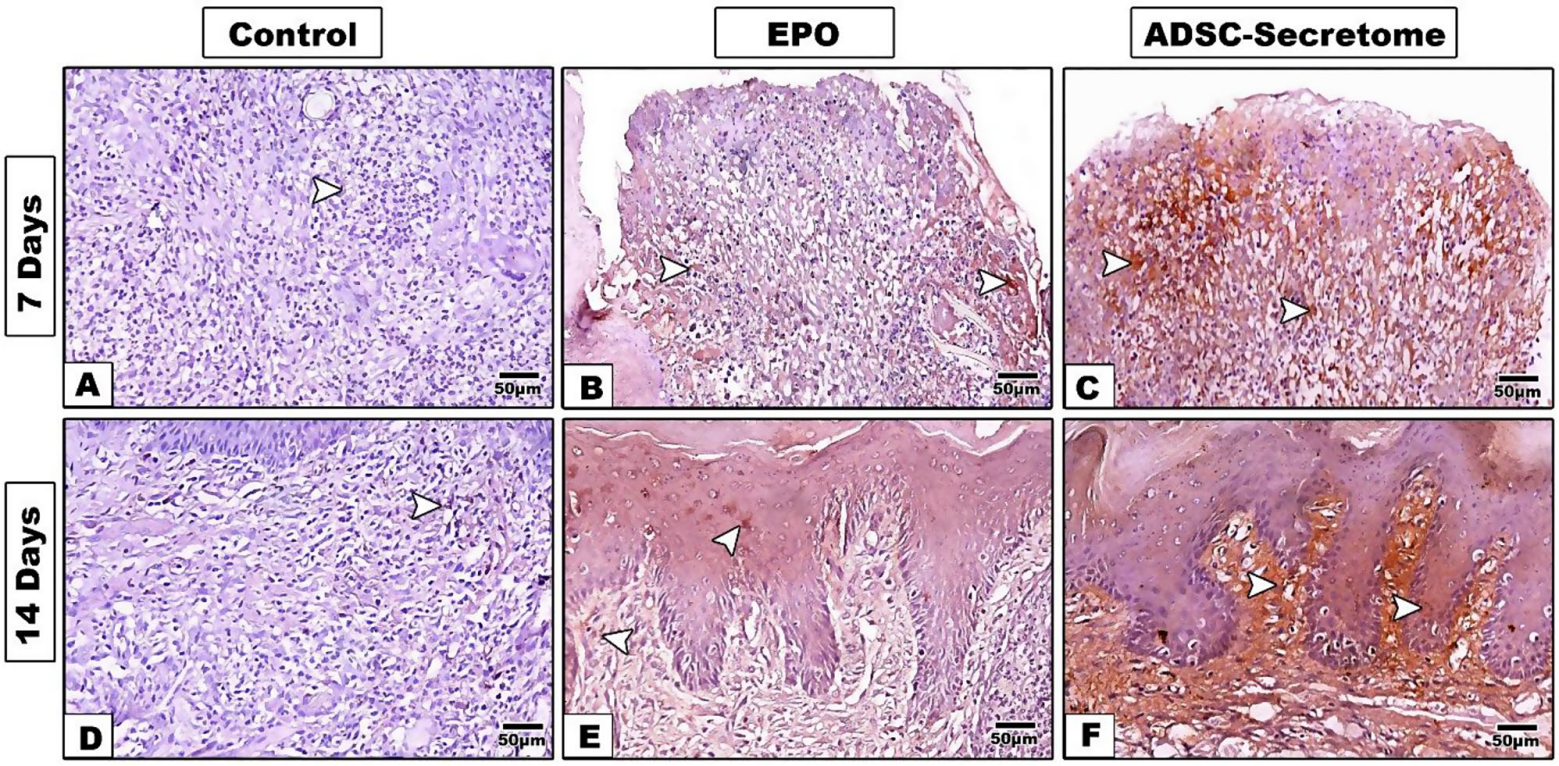
Immunostaining for CD163 antibody for different groups at 7 and 14 days (20x) **A**,** D.** control group, **B**,** E**. EPO-hydrogel group, and **C**,** F**. ADSC-Secretome group. **Arrowhead**: denotes positive brown staining areas

**Table 2:** Post Hoc Tukey test for pairwise comparison of factors affecting CD163 IHC positive staining surface area percentage

Intervention Time	Control	EPO	ADSC- Secretome
**7 days**	3.53 ± 0.86	25.77 ± 5.61 ^**a**^	27.65 ± 6.03 ^**a**^
**14 days**	12.59 ± 4.25	34.38 ± 7.11 ^**a**^	57.77 ± 11.41 ^ab^*

Data presented as Mean ± SD Significance at *p* < 0.05

^**a**^:Significance Vs control group within the same time point

^***b***^: Significance Vs EPO group within the same time point

*: Significance Vs 7 days within the same group

The positive CD163 immunohistochemical reaction was detected as brown deposits in the cytoplasm and cellular membrane (Fig. 7). CD163 expression, as a marker for M2 macrophage, denoting the anti-inflammatory activity, showed the highest levels in ADSC-Secretome group, followed by EPO, and control groups, respectively. Moreover, there was a time dependent increase at 14 days than 7 days. (Table 2) (Fig. 6B).

Two-way ANOVA analysis revealed a significant interaction between intervention and time factors on the expression of CD163 M2 macrophage marker [F [2, 24] = 8.479, *p* = 0.0016]. Therefore, a simple main effect analysis of the factors was performed. Regarding the simple main effect of the intervention factor, the pairwise comparison showed that both treatment groups; EPO and ADSC-Secretome expressed significantly higher mean values as compared to the control group at 7 days (*p* = 0.0003 and *p* < 0.0001) as well as 14 days (*p* = 0.0004 and *p* < 0.0001), respectively. However, no significant difference was detected between EPO and ADSC-Secretome groups at 7 days (*p* = 0.9975), but after 14 days, ADSC-Secretome group showed significantly higher CD163 expression (*p* = 0.0001).

Regarding the simple main effect of the time factor, no significant difference was detected between groups at the two time points, except for ADSC-Secretome group which showed significantly higher mean values at 14 days as compared to 7 days. However, control and EPO groups expressed higher mean values at 14 days than at 7 days (*p* = 0.2981, *p* = 0.351, and *p* < 0.0001 for control, EPO, and ADSC-Secretome groups, respectively).

## Discussion

Regenerative tissue engineering has been successfully implicated in the regeneration of different oral tissues, but the tongue as a vital organ hasn’t been adequately addressed in research [51]. Tongue is affected by various pathological abnormalities, including malignancies which usually require surgical resection, not only to limit the tumor growth and metastasis, but also to save the patients’ lives [52]. Unfortunately, even minor excisions affect tongue functions such as swallowing and speech, thereby adversely affecting the quality of life. Typically, these resections are reconstructed using autologous skin flaps. However, this line of treatment has many limitations regarding restoration of physiological functions [53]. The alternate strategy, despite not fully developed, is reconstructing new tissues through regenerative medicine [51]. So, the present study aimed to evaluate the regenerative potential of erythropoietin hydrogel and ADSC-Secretome in healing of mechanically induced tongue defect models and tracking the anti-inflammatory M2 macrophage through the healing process.

Animal models are widely used in experimental studies for the assessment of various disorders. It has been found that the physiological body functions of rats, as mammalians, are nearly similar to those of human beings. In addition, rats have an immune system that can withstand the induction of a variety of diseases [54]. A circular defect size of 5 mm diameter and 2 mm depth was considered a critical sized defect based on previous studies [10, 41, 55] and relying on the lack of complete spontaneous healing by the 14th day as confirmed histologically and histomorphometrically in the present study.

EPO loaded CS/β-GP/gelatin hydrogel was successfully formulated and characterized. The average particle size measured 349.5 ± 12.45 nm which is consistent with our previous study where the same EPO preparation was found to be 345.5 ± 13.01 nm [10]. This is also similar to a study by Nguyen et al. [56], who reported that the average size of EPO/magnetic nanoparticles drug delivery system was no more than 500 nm. Similarly, the average particle size for recombinant human EPO encapsulated in CS nanoparticles/TPP gel was found to range between 100 and 400 nm [57]. This moderate nanoparticle size may contribute to good regenerative potentials where according to Yue et al. [58], small nano-sized particles are faster and more uptaken by cells than micro-sized ones.

Zeta potential for the current EPO hydrogel delivery system was found to be 11.2 ± 0.90 mV. Concomitantly, in our previous study the particles expressed a zeta potential of 12.1 ± 0.87 mV [10]. Our results are also in accordance with other studies where the zeta potential of EPO-loaded trimethyl CS/TPP nanoparticles, and CS-Coated Azithromycin/Ciprofloxacin-loaded polycaprolactone nanoparticles were reported to be 11.5 ± 1.8 mV and 8–11 mV, respectively [59, 60]. According to Kumar and Dixit [61], solutions with particle zeta potential that ranges between 10 and 30 mV are considered of incipient stability. Moreover, CS was reported to decrease the particle surface charge [57].

In the current study, the control group showed deep, incompletely healed wounds, which is consistent with Xu et al. [62] who detected minor wound re-epithelization after four days of mechanical tongue ulcer induction, while after fourteen days some epithelial regeneration was observed, but without muscle re-organization. Concomitantly, in the present study, intense inflammatory infiltration was observed initially. However, after fourteen days, gradual regeneration and neovascularization with irregular epithelial coverage was detected with minimal muscle regeneration and no tongue papillae restoration. In our previous study [41], mechanical tongue wounds were examined at four, seven, and fourteen days postoperatively and similar findings were detected. These results agree with the fact that oral wound healing follows consecutive phases of inflammation, proliferation and maturation [63].

In the present study, EPO treated group showed significant decrease in defect depth and acceleration of wound healing compared to the control group. These results are in corroboration with our previous study [10], where treatment of tongue defects with EPO loaded CS/β-GP/gelatin hydrogel significantly accelerated tissue regeneration and filling of tongue defects, improved neovascularization, and reduced tissue inflammation as evidenced by the significant increase in VEGF and decrease in inducible nitric oxide synthase (iNOS) tissue levels, respectively. Similar studies conducted by Gu et al. [64] and Farid et al. [18] reported that injectable chitosan/β-GP/glycolic acid thermosensitive hydrogel loaded with EPO successfully alleviated gingival inflammation and enhanced the regeneration of periodontal tissue where EPO modulated the inflammatory mediators specially IL-1 β, IL-6 and TNF-α.

Similarly, in a study conducted by Javadmoosavi et al. [26] on the effect of EPO on the tissue-healing process, EPO-treated groups showed significant decrease in inflammatory cell density, increase in fibroblast number, neovascularization, and enhanced epithelial thickness at the wound margins compared to the control group. Consistently, mucoadhesive gel based on trimethyl chitosan containing EPO expressed better healing and epithelization of chemotherapy-induced oral mucositis in rat models [65]. Moreover, in several clinical studies conducted by Yaghobee et al. [16, 17], EPO topical application effectively enhanced palatal and gingival wound healing and reduced inflammation after free gingival graft procedures.

These positive regenerative impacts could be attributed to the ability of EPO to antagonize the proinflammatory pathways, inhibit apoptosis, and enhance neovascularization [66]. Moreover, using CS/β-GP based hydrogel, as a drug delivery system, promoted the healing process owing to its ease of application and injection into tissue spaces, gelation at body temperature, besides the sustained release of the loaded active ingredients which was optimized through gelatin incorporation that rapidly crosslinked CS with β-GP reducing gelation time [67].

In the present study, the histological sections of the ADSC-Secretome treated group showed almost no mucosal ulceration and better regenerated tissues with more regular epithelial layer with developing lingual papillae and re-organized muscle bundles. Also, there was an enhancement in neovascularization compared to EPO group which could be attributed to the fact stated by Bronckaers et al. [68] where ADSC-Secretome comprises a vast array of signaling molecules promoting angiogenesis.

These results are in accordance with our previous study [41], where ADSC-Secretome achieved promising results on the mucosal tongue defect healing; regenerating a well-organized epithelium with regular keratin layer, and the underlying connective tissue exhibited signs of muscle regeneration with more arranged collagen bundles which was attributed to the anti-inflammatory effect of ADSC-Secretome marked by downregulation of IL-6 and enhanced cellular proliferation confirmed by elevated levels of proliferating cell nuclear antibody (PCNA).

Concomitantly, in a study by Jaheen et al. [69], bone marrow stem cell-derived secretome (BMSC-Secretome) was found to enhance the healing of buccal oral ulcers showing histological re-epithelization, neovascularization, increased collagen content, and VEGF expression. ADSC-Secretome was also reported to promote the healing of full thickness skin wounds in rats showing normal epidermal regeneration after eleven days [70].

ADSC-Secretome was found to exert an anti-inflammatory effect through downregulation of activator protein-1 (AP-1), nuclear factor kappa B (NF-κB) and MAPKs signaling pathways and reduced IL-6 levels [71]. In the same context, ADSC-Secretome was found to possess anti-apoptotic influence on different cell types such as cardiomyocytes, endothelial cells, muscle cells, and epithelial cells [72], and according to Coulange Zavarro et al. [73] this antiapoptotic potential is mediated through several factors comprising TGF-β1, VEGF, HGF, Insulin-like Growth Factor-1 (IGF-1), and FGF. These factors can also recruit endothelial cells to ischemic sites enhancing neovascularization [68].

The enhanced re-epithelization could also be attributed to the facts stated by Chicharro el al. [74] where ADSCs release several growth factors that enhance re-epithelization which induce the required biological responses for tissue regeneration comprising cellular proliferation, migration, and differentiation in addition to extracellular matrix production, promotion of angiogenesis and resolution of inflammation.

However, even in ADSC-Secretome group the newly regenerated epithelium didn’t show fully developed lingual papillae and taste buds which could be attributed to their sophisticated histology which could require longer time to be fully developed which was consistent with the study conducted by Zhang et al. [75] where even after 56 days the lingual papillae and taste buds didn’t achieve full development.

Moreover, in the treated groups the underlying connective tissue only showed signs of muscle regeneration at the fourteenth day without complete restoration to normal structure, whereas epithelium and connective showed earlier full regeneration. These findings coincide with Xu et al. [62] where gingival-derived MSC (GMSCs) treated group achieved only a small percentage for positive expression of several key myogenic transcriptional factors Myf5, MyoD, and PAX7. In our previous studies, the regeneration of tongue musculature was also postponed to later stages [10, 41] which indicates that muscle repair might require longer time than mucosal epithelium or connective tissue regeneration.

In response to environmental influences, macrophages undergo modulation between the pro-inflammatory M1 and the anti-inflammatory M2 phenotypes [43]. CD163 is an M2-macrophage specific antigen involved in tissue remodeling and release of immune-modulatory mediators such as IL-4, IL-10, IL-13, and TGF-β [76]. It is considered to be highly specific for M2 macrophage phenotype according to literature [77–79].

In the current study, EPO and ADSC-Secretome treatments significantly enhanced the anti-inflammatory effect through promoting the upregulation of M2 macrophage phenotype as evidenced by upregulation of CD163 marker. Concomitantly, in a study by Li et al. [80], EPO hydrogel, through suppression of ROS levels, successfully shifted macrophage polarization from M1 to M2 enhancing tissue regeneration in periodontitis where M1 macrophage markers (IL-1β, IL-6, and TNF-α) were down-regulated while M2 macrophage markers (IL-10, Arg-1, and TGF-β) were upregulated. This modulation decreases the levels of matrix metalloproteinase-9 (MMP-9) and cyclooxygenase-2 (COX-2) favoring healing through restoration of homeostasis [18].

Kruger et al. [81] declared that ADSC-Secretome can restore the inflammatory balance through promotion of M2 macrophage phenotype and increasing the percentage of cells expressing CD206, CD163, and IL10. In the same context, Li et al. [82] found that ADSC-exosomes can control M1/M2 macrophage phenotypic shift promoting healing through regulation of miR-451a macrophage migration inhibitory factor.

In contrast to our results, a study on the impact of intraperitoneal EPO injection on ischemic wound healing in rat models found that it enhanced tissue healing in the early stages, but later it hindered regeneration which was attributed to high hemoglobin and hematocrit values, that may have affected the efficiency of microcirculation [83]. Regarding the therapeutic influence of MSC-Secretome, Conforti et al. [84] informed that, compared to native whole cells, MSC-Secretome didn’t achieve the required immunomodulation which they attributed to lack of cell to cell contact required to regulate inflammatory cells proliferation and function. Moreover, in a model of acute renal injury studied by Gabr et al. [85], intravenous administration of BMSC-Secretome didn’t show significant regenerative effects. Controversies between the results of different studies of different treatments are largely accredited to different tissues, injury models, doses and formulations of different therapeutic agents, delivery systems, preparation protocols, evaluation periods, and the implicated assessment parameters.

From the limitations of the current study is the lack of comparison of different EPO and ADSC-Secretome concentrations that may have influenced their therapeutic potentials. Moreover, in the present study, only histological and immunomodulatory analysis were performed which doesn’t fully explain the different biological processes yielding tissue regeneration. Only one marker for M2 macrophage was used in the present study which could have been supported by other markers, however CD163 is considered to be highly specific for M2 macrophage. In addition, the composition of ADSC-Secretome, which comprises a large pool of diverse biological molecules, wasn’t investigated.

So further future studies are required to address these limitations. More analysis for the different phases of the tissue healing process is needed to elucidate the typical underlying tissue repair mechanisms. Moreover, detailed compositional analysis of the secretome accompanied by long term follow up assessment is mandatory to ensure its safety before translation to human clinical studies.

## Conclusion

Eventhough ADSC-Secretome showed better results, both EPO-hydrogel and ADSC-Secretome showed promising and comparable results in the treatment of tongue wounds with significant reduction in defect depth and significant upregulation of the anti-inflammatory M2 macrophage phenotype suppressing inflammation thus enhancing healing and regeneration of tongue tissues. Therefore, the use of ADSC-Secretome is recommended as a cell free therapy overcoming the concerns associated with the use of stem cells and at the same time, EPO-hydrogel is presented as a potential, affordable, and easily obtained therapy.

## Data Availability

All the produced data is included in this article.

## Abbreviations

EPO: Erythropoietin
ADSCs: Adipose derived stem cells
β-GP: β-sodium glycerophosphate
IL-1β: Interleukin-1β
IL-6IL-6: Interleukin-6
TNF-α: Tumor necrosis factor-alpha
MAPKs: Mitogen-activated protein kinases
PI3K: Phosphoinositide 3 kinase
TPP: Tripolyphosphate
VEGF: Vascular endothelial growth factor
MSCs: Basic fibroblast growth factor
TGF-β: Transforming growth factor- beta
KGF: Keratinocyte growth factor
HGF: Hepatocyte growth factor
Fpr2: Formyl peptide receptors
Gpr18: G protein
TLR: Toll-like receptors
Egr2: Early growth response gene-2
MU-ACUC: Mansoura University animal care and use committee
MERC: Medical experimental research center
TPP: Tripolyphosphate
NaOH: Sodium hydroxide
DLS: Dynamic Light Scattering
DMEM: Dulbecco’s modified eagle medium
FBS: Fetal bovine serum
PBS: Phosphate buffered saline
FITC: Fluorescein isothiocyanate
H&E: Hematoxylin and eosin
ANOVA: Analysis of variance
iNOS: Inducible nitric oxide synthase
PCNA: Proliferating cell nuclear antibody
IGF-1: Insulin-like Growth Factor-1
AP-1: activator protein-1
NF-κB: Nuclear factor kappa B
ROS: Reactive oxygen species
MMP-9: Matrix metalloproteinase-9
COX-2: Cyclooxygenase-2
